# Expression of Cry2Aa, a *Bacillus thuringiensis* insecticidal protein in transgenic pigeon pea confers resistance to gram pod borer, *Helicoverpa armigera*

**DOI:** 10.1038/s41598-018-26358-9

**Published:** 2018-06-11

**Authors:** Shweta Singh, Nikhil Ram Kumar, R. Maniraj, R. Lakshmikanth, K. Y. S. Rao, N. Muralimohan, T. Arulprakash, K. Karthik, N. B. Shashibhushan, T. Vinutha, Debasis Pattanayak, Prasanta K. Dash, P. Ananda Kumar, Rohini Sreevathsa

**Affiliations:** 1 ICAR-National Research Centre on Plant Biotechnology, Pusa Campus, New Delhi, India; 2 Division of Biochemistry, ICAR-Indian Agricultural Research Institute, Pusa Campus, New Delhi, India; 3 ICAR-Indian Institute of Rice Research, Hyderabad, India; 4 Department of Crop Physiology, University of Agricultural Sciences, GKVK, Bangalore, India

## Abstract

Pigeon pea is an important legume infested by a plethora of insect pests amongst which gram pod borer *Helicoverpa armigera* is very prominent. Imparting resistance to this insect herbivore is of global importance in attaining food security. Expression of insecticidal crystal proteins (ICP) in diverse crops has led to increased resistance to several pests. We report in this paper, expression of Cry2Aa in transgenic pigeon pea and its effectiveness towards *H*. *armigera* by employing *Agrobacterium*-mediated *in planta* transformation approach. Approximately 0.8% of T_1_ generation plants were identified as putative transformants based on screening in the presence of 70 ppm kanamycin as the selection agent. Promising events were further recognized in advanced generations based on integration, expression and bioefficacy of the transgenes. Seven T_3_ lines (11.8% of the selected T1 events) were categorized as superior as these events demonstrated 80–100% mortality of the challenged larvae and improved ability to prevent damage caused by the larvae. The selected transgenic plants accumulated Cry2Aa in the range of 25–80 µg/g FW. The transgenic events developed in the study can be used in pigeon pea improvement programmes for pod borer resistance.

## Introduction

Pigeon pea is an important grain legume in Asia, Africa and parts of Latin America^1,2^. In India, pigeon pea is grown in 3.8 million hectares^3,4^ contributing to 90% of global production. High level of protein in pigeon pea makes it an important component of diet especially amongst the Indian vegetarian population. Despite improved production of pigeon pea to 4.85 million tonnes over the last decade, there has been stagnation in the yield per hectare^2^. One of the major reasons for this is its susceptibility to a lepidopteran pest, gram pod borer (*H*. *armigera*)^5^. It is the most difficult pest to control with high fecundity and strong migratory behaviour resulting in approximately 85% of yield loss. The herbivore apparently is responsible to cause losses up to or more than US $300 million annually^6^. The wide host range, high degree of migration, indiscriminate pesticide application by farmers and innate ability of the insect to quickly develop resistance to insecticides have made it attain the status of a key pest^7,8^. Furthermore, screening of more than 14,000 accessions of cultivated pigeon pea has revealed moderate or low levels of resistance towards the insect^9,10^. Therefore, incorporating the trait for pod borer resistance is an integral part of pigeon pea crop improvement.

One of the effective strategies to manage gram pod borer is transgenic expression of *Bacillus thuringiensis* insecticidal crystal proteins (ICPs), also known as delta-endotoxins^11,12^. A number of *cry* genes with effectiveness against insect pests are incessantly being identified by scientists worldwide for their introgression into crop plants through transgenesis^13^. Individual Cry toxins are usually toxic to only a few insect species within an order and receptors on midgut epithelial cells have been shown to be critical determinants of Cry specificity. The increasing acreage of the transgenic crops harboring these *Bt* ICPs is demonstration enough for the acceptance and success of the technology^14^. There have been several reports about the use of various *Bt* ICPs in pigeon pea to manage pod borer^15–18^. However, several transgenic events harboring a range of effective ICP genes need to be developed so that superior events with commercialization potential and sustainability under field conditions can be identified.

In addition to Cry1 series of ICPs, it is pertinent to assess the ability of another ICP, Cry2Aa, for its efficacy against *H*. *armigera* in pigeon pea. In addition, utilizing a non-tissue culture-based *in planta* approach^19^ for the development of stable and effective events can be an added advantage due to the recalcitrance of pigeon pea to tissue culture. Earlier, *cry2Aa* has been tested and proven effective against *H*. *armigera* in transgenic rice, chickpea and pigeon pea^17,20–22^. Transgenic plants with *cry2Aa* can consequently be used as an additional strategy for the development of insect resistant pigeon pea^17,23–25^ to tackle the devastating herbivore. Our group is also actively involved in the development of pigeon pea transgenics with other novel *Bt* ICPs^26^. Since the mode of action of *cry2Aa* is different when compared to *cry*1 series of *Bt* ICPs^17^, stacking transgenic crops expressing both the genes can be a viable approach for delaying evolution of resistance to cry toxins by *Helicoverpa armigera*. In this direction, the purpose of the present study was to demonstrate the utility of *cry2Aa* gene in transgenic pigeon pea against *H*. *armigera* and also reiterate the applicability of an apical meristem-targeted tissue culture-independent *in planta* transformation approach for pigeon pea improvement.

## Results

### Development and selection of transformants in pigeon pea carrying *cry2Aa* gene

**Figure 1:**
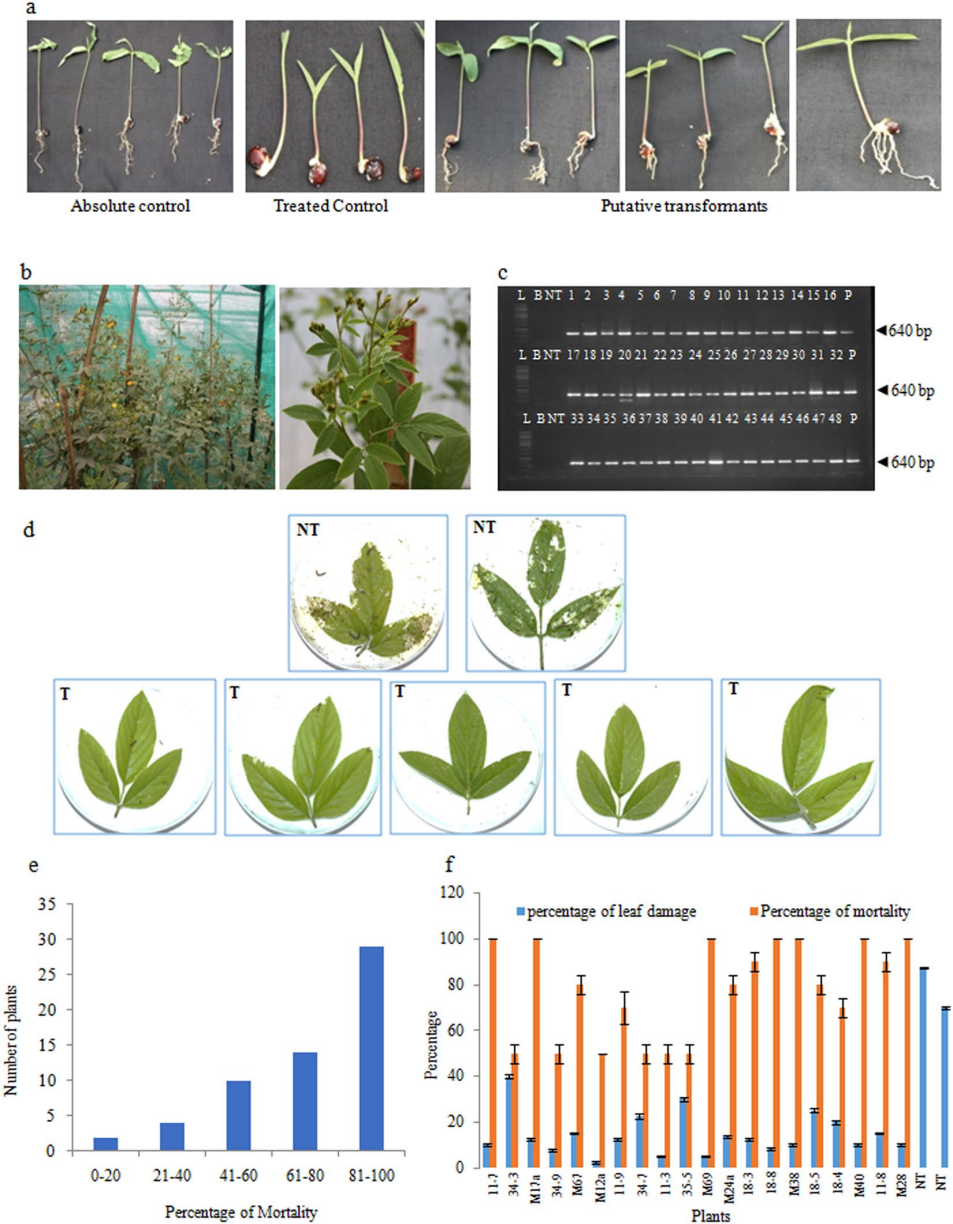
Analysis of T_1_ generation plants. (**a**) Screening for the selection of putative transformants grown in quartz sand under the selection pressure of 70 ppm kanamycin; (**b**) putative transgenic plants established in the greenhouse; (**c**) PCR analysis of T_1_ generation plants for the amplification of a 640 bp *cry2Aa* gene fragment (L: 1 kb ladder, B: template blank, NT: non transgenic, lane 1–48 = representative samples of putative transformants, P: plasmid); (**d**) Performance of putative transformants *vis- á- vis* non transgenic plants in the *in vitro* leaf bioassay against neonate larvae of *Helicoverpa armigera* (NT: non transgenic plants; T: putative transformants); (**e**) frequency distribution of the plants based on percentage mortality of *H. armigera* in the bioassay; (**f**) a representative histogram depicting the variation in the performance of T_1_ generation plants in the *in vitro* bioassay.

Primary transformants of pigeon pea cv. Pusa 992 were developed using the apical meristem-targeted *Agrobacterium*-mediated *in planta* transformation strategy^19^. About 50 seedlings were subjected to *in planta* transformation of which 28 individual T_0_ plants (primary transformants) established in the green house that grew normally, flowered and set seeds. Since the transformants developed by *in planta* transformation strategy are chimeras in T_0_ generation, screening of T_1_ generation plants for the identification of putative transformants^27^ is essential. In the present study, preliminary screening of the seedlings on 70 ppm kanamycin identified 59 putative transformants in T_1_ generation (Fig. 1a) that grew normally and established in the greenhouse (Fig. 1b). This accounted for 0.8% of the total number of seeds that were subjected to screening. Further to confirmation of the presence of T-DNA by PCR analysis, (Fig. 1c), leaf bioassays against neonate larvae of *H*. *armigera* revealed significant variability in larval mortality and the extent of damage to the leaves (Fig. 1d). There was diversity in the response of T_1_ generation plants to the larval challenge which varied between 0–100% larval mortality (Fig. 1e); moreover, the plants that showed high mortality exhibited less leaf damage (Fig. 1f). Based on the stringent efficacy evaluation, 29 T_1_ generation plants (about 49% of the total putative transformants selected on kanamycin) were selected as promising. These experimental evidences provided comprehensible evidence about the transgenic nature of the plants. The selected plants were advanced to T_2_ generation for the analysis of stability in T-DNA integration, inheritance as well as sustained efficacy.

### Analysis of the transgenic plants for stable integration and inheritance of T-DNA

**Figure 2:**
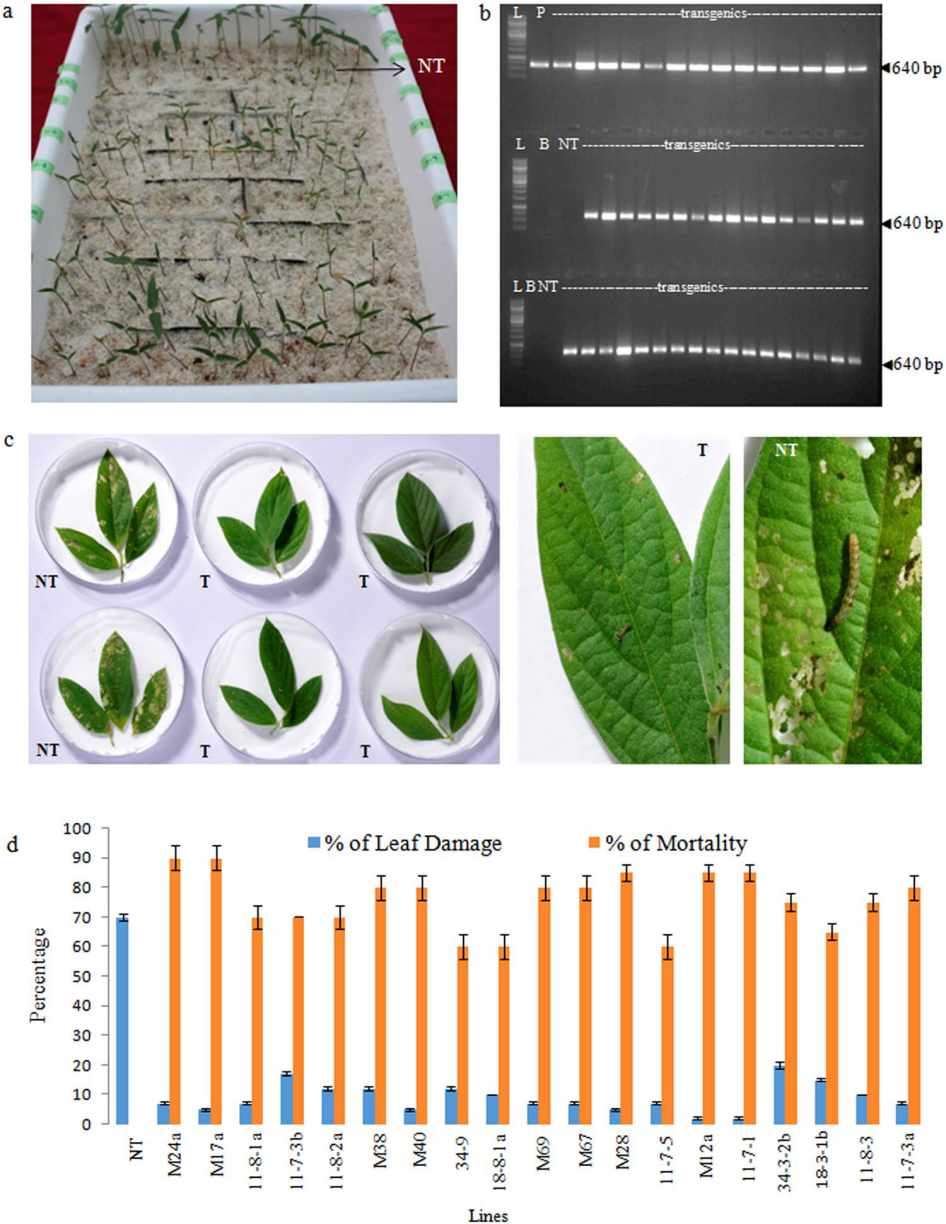
Analysis of transgenic plants in T_2_ generation. (**a**) Stability in the resistance response of the progeny of selected T_1_ plants on 70 ppm kanamycin (NT: non transgenic plants); (**b**) PCR analysis of T_2_ generation plants for the amplification of a 640 bp *cry2Aa* gene fragment (L: 1 kb ladder, B: template blank, NT: non transgenic; P: plasmid); (**c**) *in vitro* leaf bioassay to depict the performance of transgenic plants *vis- á- vis* non transgenic plants (NT: non transgenic plants; T: putative transformants); (**d**) histogram portraying the performance of individual transgenic lines to deliberate challenging of the larvae of *H. armigera* in terms of percent mortality of the larvae and extent of leaf damage caused by the larvae.

**Figure 3:**
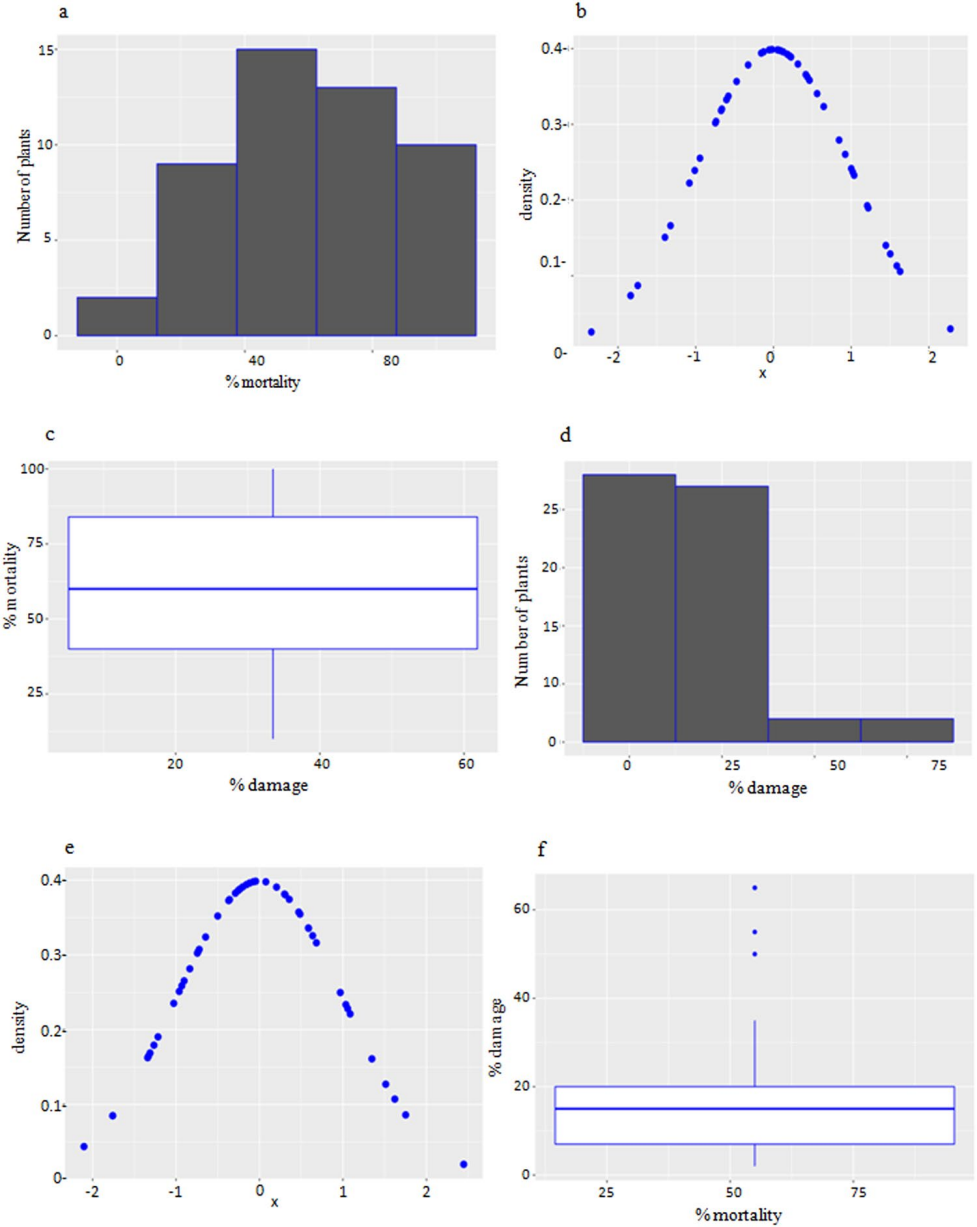
Analysis of the performance of T_2_ generation plants against *H. armigera* in the leaf bioassays. (**a**) Histogram depicting the performance of T_2_ generation plants in the *in vitro* leaf bioassay with respect to mortality of *H. armigera* larvae; (**b**) bell curve demonstrating the skewed distribution of percent mortality; (**c**) comparative box whisker plot elucidating the performance spread of transgenic plants depicting improved mortality of the larvae (Min. 10.00; 1st Qu. 40.00; Median 60.00; Mean 59.82; 3rd Qu. 84.00; Max. 100.00); (**d**) histogram depicting the percent damage caused by *H. armigera* on the leaves; (**e**) bell curve demonstrating the skewed distribution of percent damage; (**f**) Comparative box whisker plot depicting the extent of damage caused by the larvae (Min. 2.00; 1st Qu. 7.00; Median 15.00; Mean 17.12; 3rd Qu. 20.00; Max. 65.00).

**Figure 4:**
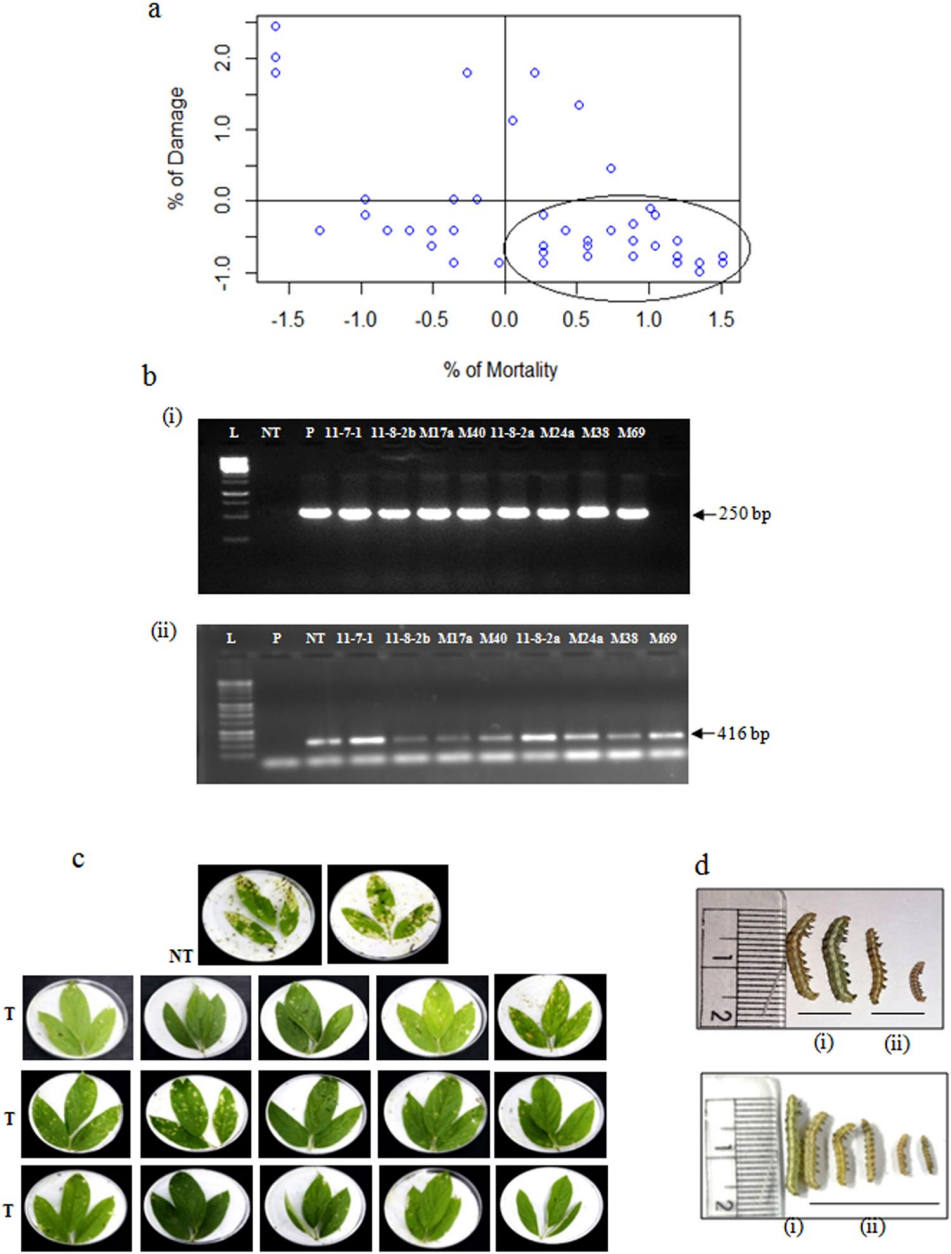
(**a**)Z distribution analysis for two parameters, percent mortality of *H. armigera* larvae and % damage caused by the larvae in the *in vitro* leaf bioassay; (**b**) sqRT-PCR analysis in the selected T_2_ generation transgenic plants for transcript accumulation of (i) *cry2Aa* gene (ii) *18s* *rRNA* gene as an internal control (L: ladder; NT: non transgenic; P: plasmid); (**c**) depiction of the performance of selected transgenic plants of T_2_ generation in the *in vitro* leaf bioassay against deliberate challenging of *H. armigera*; (**d**) Larval morphology at the end of *in vitro* leaf bioassay after feeding on (i) non transgenic plants; (ii) transgenic plants (event, M40).

Ability of the progeny of the selected 29 T_1_ generation plants to survive and grow on 70 ppm kanamycin (Fig. 2a) demonstrated that the T-DNA was stably integrated in the genome of the transgenic plants. Further molecular and bioefficacy analysis to confirm the inheritance of T-DNA was carried out with these plants that survived on kanamycin selection. The first molecular evidence for the stability of T-DNA was demonstrated by PCR analysis for the gene of interest *cry2Aa* and the expected amplicon of 640 bp was observed in all the surviving transgenics (Fig. 2b). *In vitro* leaf bioassays of the established T_2_ generation plants against deliberate challenging with neonate larvae of *H*. *armigera* demonstrated their superior bioefficacy (Fig. 2c-representative picture) in terms of higher larval mortality and lesser leaf damage (Fig. 2d). The bioassays conclusively demonstrated that the selected transformants not only had the *cry2Aa* gene integrated stably in their genome (Fig. 3a,d) but also their greater ability to resist the insect herbivory as depicted explicitly by the bell curve for two parameters *viz*., insect mortality and leaf damage (Fig. 3b,e). Further, it was observed that, >75% of the plants in the T_2_ generation demonstrated <20% leaf damage with synchronised mortality (Fig. 3c,f). However, based on Z distribution analysis (Fig. 4a) for the two parameters, larval mortality and extent of leaf damage, 15 events (in the 1^st^ quadrant) were identified as superior. Further, randomly selected superior events concomitantly confirmed accumulation of *cry2Aa* gene transcripts (Fig. 4b) implying that the performance of the events in the bioassay was because of the expression of the integrated *cry2Aa* gene. Ability of the selected plants to combat the herbivore was reiterated by their performance after challenging the leaves with *H*. *armigera* (Fig. 4c-representative photograph). Variation in larval morphology in terms of drastic reduction and mortality upon feeding on some of the transgenics was clearly evident at the end of leaf bioassay (Fig. 4d). However, larvae that fed on the non transgenic plants depicted normal morphology and development (Fig. 4d). Based on the molecular and bioefficacy analysis, 15 events, i.e., approximately 25% of the earlier identified T_1_ generation plants (M24a, M17a, 11-8-1a, 11-7-3b, 11-8-2a, M38, M40, M69, M67, M28, M12a, 11-7-1, 34-3-2b, 11-8-3, 11-7-3a) were advanced for further analysis in T_3_ generation.

**Figure 5:**
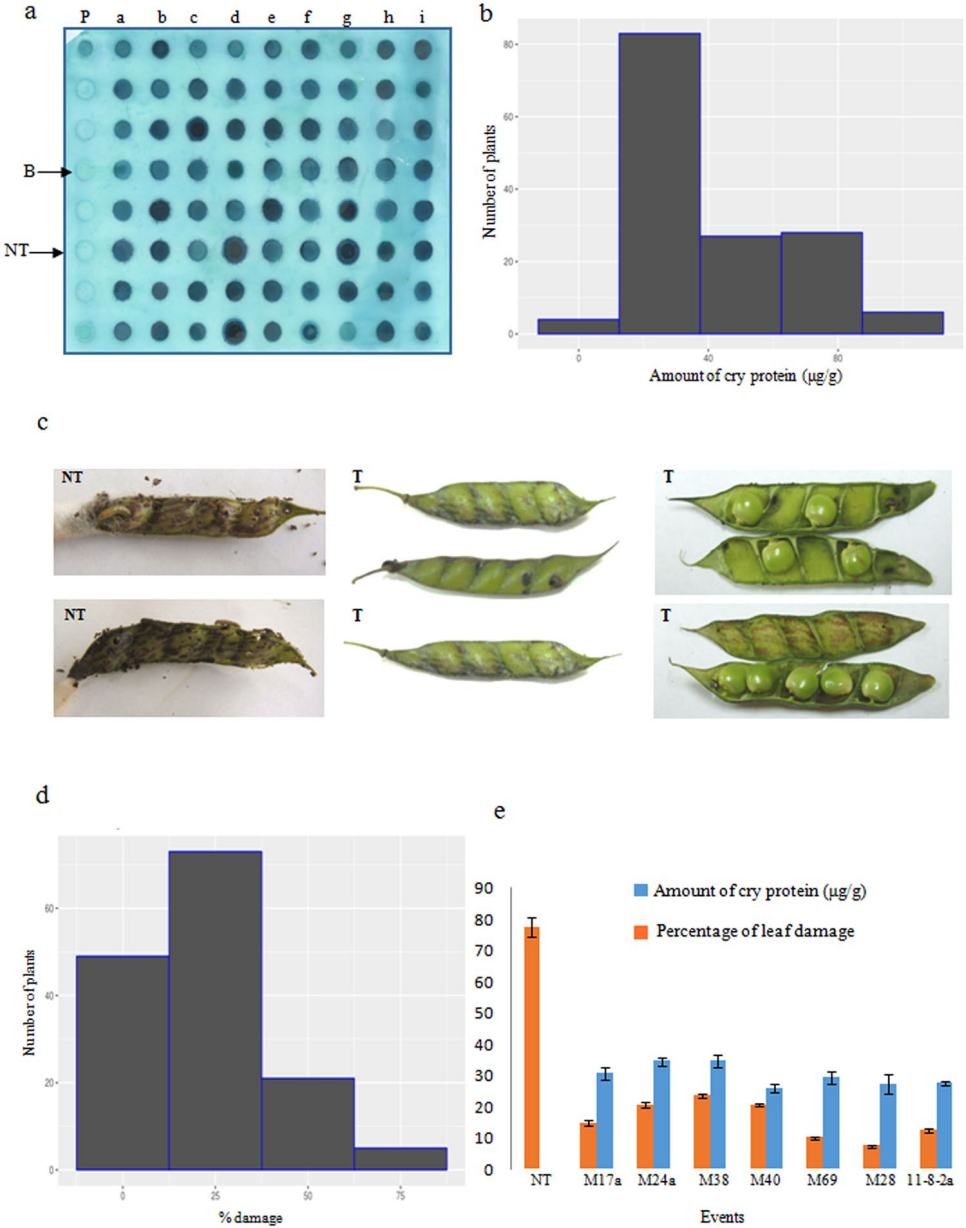
Analysis of T_3_ generation plants. (**a**) High throughput dot blot analysis (representative blot) showing the stable expression of Cry2Aa in different transgenic plants *vis- á- vis* non transgenic (P: 20 ng purified Cry2Aa protein; B: blank; NT: non transgenic; a-i: total protein extracts from different transgenic lines comprising of 8 plants per line); (**b**) Histogram illustrating the expression levels of Cry2Aa protein in µg/g FW across various transgenic plants; (**c**) Performance of the transformants *vis-* *a-* *vis* non transgenic plants against deliberate challenging of *H. armigera* in an *in vitro* pod bioassay (NT: non transgenic plants; T: transformants); (**d**) Histogram illustrating the percent damage across various transgenic plants following pod bioassay; (**e**) Performance of the selected plants in pod bioassay *vis- á- vis* Cry2Aa protein accumulation (µg/g FW).

In the advanced T_3_ generation, dot blot analysis for Cry2Aa expression was used as an initial proof to assess the stability in the transgene expression in the selected events. The selected events demonstrated strong signals not only indicating high levels of protein but also uniformity in expression across the progeny of the selected transformants (Fig. 5a). Quantitative analysis of the dot blot showed that Cry2Aa expression varied between ~7 µg/g FW and ~96 µg/g FW of leaf tissue. Maximum number of transgenic plants expressed in the range of 25–50 µg/g FW of the Cry2Aa protein (Fig. 5b). Based on this observation, stability in efficacy of these plants against *Helicoverpa* was analysed in well-developed pods and the extent of damage caused by the 2^nd^ instar larvae was considered as the parameter for analysis. Study of the performance of selected transgenic plants demonstrated a clear constancy in the ability of the plants to resist the attack of the larvae with reduced damage (Fig. 5c). Damage was seen to be skewed to the left as maximum number of plants demonstrated 0 to 25% leaf damage clearly indicating that the damage was well under control (Fig. 5d). Segregation analysis carried out with the selected events provided additional proof for the stable integration and inheritance of the T-DNA (Table 1). The analysis depicted that majority of the selected events followed the expected 3:1 segregation ratio indicating T-DNA stability. However, among the various promising events that were subjected to evaluation, 7 events (M17a, M24a, M38, M40, M69, M28, 11-8-2a) with consistent Cry2Aa protein expression and less damage when challenged with *H*. *armigera* were identified as promising. The selected events showed coherence in both the parameters under analysis as they not only showed higher protein expression in the range of 26–34 µg/g FW but also efficiently resisted the attack of the pest by demonstrating reduced damage (7.5–23%) (Fig. 5e). Comparison of means by two tailed ‘t’ test with unequal sample sizes, indicated that the selected transgenic plants were significantly superior with respect to the accumulation of Cry2Aa protein (t = 35.778; P < 2.2e^−16^). Similarly, significant difference was also seen with respect to leaf damage by *H*. *armigera* (t = −30.883; P 5.54e^−14^) demonstrating the ability of the transgenic plants to resist the herbivore attack (Table 2).

**Table 1:** Transgene inheritance analysis in the selected transgenic events based on bioassay against *H*. *armigera*.

Sl. No	Event identity	Total number of plants	Positive plants	Negative plants	χ^2^	P
1	11-7-3a	13	9	4	0.23	0.632
2	11-8-2a*	17	11	6	0.96	0.327
3	M40a	6	1	5	10.89	<0.001
4	M17a*	4	0	4	12.00	<0.001
5	M17b	5	0	5	15.00	<0.001
6	M24a*	4	3	1	0.00	1
7	M27	8	2	6	10.67	<0.001
8	M40*	12	7	5	1.78	0.182
9	M40b	8	6	2	0.00	1
10	M69*	23	18	5	0.13	0.718
11	11-8-1	14	6	8	7.71	0.006
12	11-8-2b	11	6	5	2.45	0.116
13	11-7-3b	2	0	2	6.00	0.014
14	M38*	2	0	2	6.00	0.014
15	M28*	7	7	0	2.33	0.127

^*^Seven selected superior events.

Segregation analysis was calculated based on the performance of transgenic plants in the pod bioassay. The plants that showed <25% pod damage were considered as positive.

**Table 2:** Comparison between the transgenic plants of T_3_ generation *vis- á- vis* non-transgenic plants with respect to Cry2Aa protein content and pod damage in the insect bioassay.

	n	Protein Content		% Leaf Damage	
		Mean ± SD	Range	Mean ± SD	Range
Transgenics	50	29.34 ± 5.79	18.35–44.28	9.49 ± 4.96	1.25–20
Non-Transgenic plants	12	0 ± 0	0	77.5 ± 7.22	70–90
T-test		35.778		−30.883	
P-values		<2.2e^−16^		5.54e^−14^	

### Molecular characterization of the transgenic events for transgene integration and inheritance

**Figure 6:**
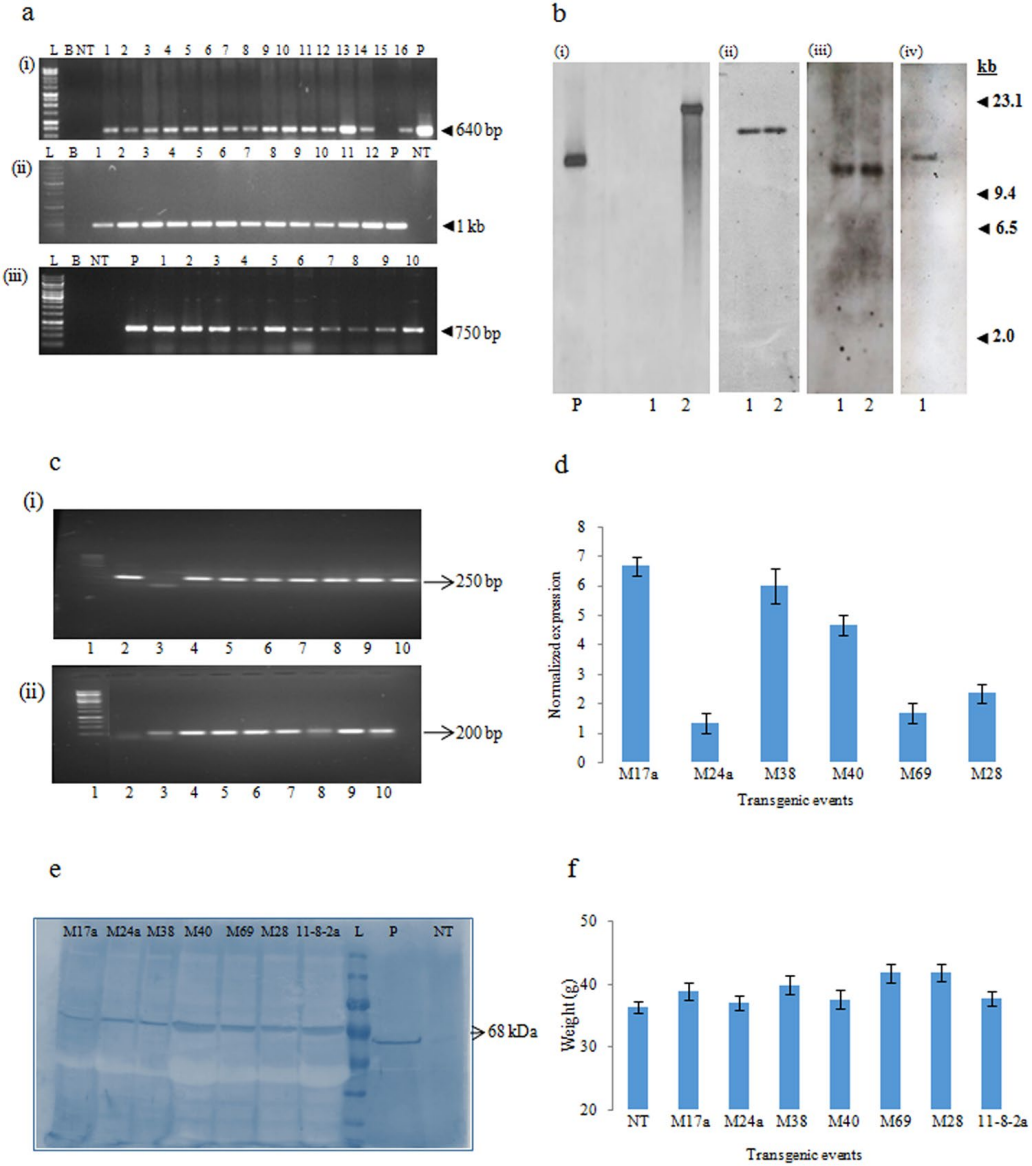
Molecular analysis of the selected T_3_ generation transgenic plants. (**a**) PCR analysis for the amplification of *cry2Aa* (i and ii) and (iii) *nptII* gene fragments in the transgenic plants *vis- á- vis* non transgenics; (**b**) Genomic Southern analysis of transgenic pigeon pea events: 15 µg of genomic DNA was digested with *HindIII* and probed with a 640 bp DIG labelled *cry2Aa* gene fragment (i) M17a (Lane P: pBinAR plasmid-250 pg; Lane 1: DNA from non transgenic plant; Lane 2: DNA from event M17a); (ii) M40 (Lane 1: DNA from M40–1 and Lane 2: DNA from 40–2); (iii) M69 (Lane 1: DNA from M69-1and Lane 2: DNA from M69-2); (iv) 11-8-2a; (**c**) sqRT-PCR analysis in the selected T_3_ generation transgenic plants for transcript accumulation (i) amplification of 250 bp *cry2Aa* gene fragment; (ii) amplification of a 200 bp actin gene fragment as an internal control (Lane 1: ladder; Lane 2: plasmid; Lane 3: non transgenic plant; Lanes 4–10: selected transgenic plants (M17a, M24a, M38, M40, M69, M28, 11-8-2a); (**d**) qRT-PCR analysis of six selected events for the expression of *cry2Aa* gene. *Initiation Factor 4α* gene was used as an internal control and ∆Ct values were calculated using difference in the Ct mean of the target gene and the reference gene. X axis represents different transgenic events and Y axis represents normalized expression of the transgene; (**e**) Western blot analysis for the expression of Cry2Aa in the selected T_3_ generation plants (Lane L: ladder; Lane NT: total protein extract from the non transgenic plant; Lane P: purified Cry2Aa [30 ng]); (**f**) pod yield at maturity in different selected transgenic plants *vis- á- vis* non transgenic plants.

Comprehensive molecular characterization of the 7 superior events was carried out for unambiguous demonstration of stable integration and inheritance of T-DNA. PCR analysis using primers specific for both *cry2Aa* and *nptII* (Fig. 6a) demonstrated their presence in the genomic DNA of the selected events. Further, genomic Southern analysis revealed that the T-DNA was integrated as a single copy in four of the superior events, M17a (Fig. 6b[i]), M40 (Fig. 6b [ii]), M69 (Fig. 6b[iii], and 11-8-2a (Fig. 6b [iv]). To corroborate integration, expression and efficacy analyses, all the selected transgenic plants when further assessed for transcript accumulation by both sqRT-PCR (Fig. 6c; Supplementary fig. S2) and qPCR (Fig. 6d) showed varied levels of *cry2Aa* expression in different events. The conclusive evidence for the expression of Cry2Aa in all the selected transgenic events was further supported by western blot analysis (Fig. 6e) using Cry2Aa-specific antibody reiterating that the observed superior bioefficacy in these events against *H*. *armigera* was due to the integrated *cry2Aa* transgene. Further, absence of variation in the pod yield in selected transgenic plants *vis- á- vis* non transgenic plants at maturity demonstrated that the integrated T-DNA did not result in any unintended effects either due to the position of the T-DNA or due to the accumulation of Cry2Aa protein (Fig. 6f).

## Discussion

Development of insect resistance in crop species using biotechnological tools has gained prominence over the past three decades^28,29^. Since the advent of genetic engineering, the major candidate proteins for achieving insect resistance have been the ICPs (insecticidal crystal proteins) and *Bt* ICPs in particular^30^. Of the very many insect pests, *H*. *armigera*, a lepidopteran pest is one of the most serious and widespread pests of pigeon pea^31^. Increasing use of toxic pesticides^32^ as well as development of resistance by pests^29^ has led to the continued quest for alternate approaches to tackle *H*. *armigera*. Modern genomic tools such as molecular markers and candidate genes associated with resistance offer the possibility of facilitating pigeon pea breeding towards improved biotic stress resistance. However, absence of known sources of resistance and limited genetic variability in the cultivated germplasm^1^ has minimized the utility of conventional breeding approaches to address this problem. Insect resistant transgenic plants, therefore, are considered to be an ideal seed borne solution^32,33^.

Effectiveness of *Bt* ICPs in the control of pod borer has been demonstrated earlier in pigeon pea^15–18,34,35^. However, potential events arising from various strategies need to be assessed to identify a prospective product with an ability to sustain the devastating herbivore under field conditions. This necessitates the use of a variety of genes and strategies for the development of viable transgenic events. The recalcitrance of pigeon pea to tissue culture and reduced ability in the development of transformants has resulted in researchers adopting non-conventional approaches for the development of transformants in this crop^15,16,18,19^. In the present study, a tissue culture independent apical meristem-targeted *in planta* transformation that was standardized earlier by our group was used to develop transformants in pigeon pea^19^. The strategy mainly deals with the *in planta* inoculation of the shoot apical meristem and allowing them to grow *ex vitro*. The progeny of the chimeric primary transformants are required to be screened for the identification of putative transformants in the T_1_ generation. The major advantage of this transformation strategy as demonstrated in our earlier studies is the ability to develop a large number of primary transformants^27^. This necessitates the need of a high throughput yet stringent selectable marker-based screening for the initial identification of putative transformants. In this study, quartz sand-based screening of a large number of T_1_ generation seedlings in the presence of the selection agent kanamycin (70 ppm)^27^ resulted in the identification of 59 putative transformants. This selection strategy not only allowed the identification of putative transformants but also contributed towards selection of transgenics expressing the selectable marker gene in higher levels^27^ under the given selection pressure. The putative transformants were advanced with an assumption that correlation would exist between the expression of selectable marker gene as well as the target gene. The major emphasis in the present study was in the identification of promising pigeon pea events depicting increased Cry2Aa expression and superiority in their efficacy against pod borer, *H*. *armigera*. Both leaf and pod-level rigorous *in vitro* bioassays performed in successive generations aided in the identification and selection of events with 80–100% larval mortality and <5–10% leaf or pod damage. This was substantiated by in-depth analysis of the bioefficacy studies carried out in the respective generations. Based on this, 29 and 15 events were selected at the end of T_1_ and T_2_ generation with these events demonstrating categorical evidences of T-DNA integration.

It was observed that, with the advancement of generations, the performance of the overall population of plants in T_2_ and T_3_ generations improved towards resisting *H*. *armigera*. Further proof of the stability of the transgene was obtained by segregation analysis which showed that most of the selected plants followed the Mendelian 3:1 ratio. Additionally, majority of the plants accumulated Cry2Aa protein that was effective against the pest reiterating the stability of the integrated T-DNA and promising events expressing in the range of 26–34 µg/g FW. Though the expression levels of *Bt* ICPs can vary across crops and genes^15,17^, the quantity estimated in the study was on par with other studies where Cry protein expression has been provided^17^. Meticulous analysis for the confirmation of integration, expression and efficacy resulted in the identification of 7 superior events, which are now being advanced to identify a viable commercial event through event selection trials. Though studies have demonstrated the ability of different *Bt* ICPs to combat *H*. *armigera*, very few depict the stability of the transgene in advanced generations^15,34^. Moreover, since utility of *cry2Aa* in pigeon pea has not been widely proved, except for a single study^17^, this study is the first of its kind to prove the stability in the expression and performance of the transgenic pigeon pea plants up to T_3_ generation.

We provide evidence for the usefulness of the apical meristem-targeted *in planta* protocol not only in successful transformation of an economically important crop like pigeon pea but also the ability to develop a large number of superior events. Stable events with high expression and efficacy of Cry2Aa have been identified and advanced till T_3_ generation. The utilized approach as well as the set of promising events can be an effective contribution to the fraternity trying to effectively mitigate the devastating herbivore through transgenesis.

## Materials and Methods

### Development of primary transformants and identification of putative transformants

Primary transformants in pigeon pea were developed following the apical meristem-targeted tissue culture independent *in planta* transformation protocol^19^ in the cv. Pusa 992, which is a susceptible genotype to *H*. *armigera*. The 1.8 kb *cry2Aa* gene used in the study was synthesized and validated at ICAR-NRCPB. The gene was sub-cloned in pBinAR (Supplementary Fig. S1a), mobilized into Agrobacterium strain EHA105 and used for transformation. The T_1_ seeds from the primary transformants were harvested and analysed subsequently for the selection of putative transformants.

Screening of T_1_ transformants was carried out in the presence of 70 ppm kanamycin^27^. Four day old germinated seedlings were soaked in 70 ppm kanamycin for 4 h at room temperature and transferred to quartz sand. These seedlings were grown under greenhouse conditions for 10 days and supplemented with ¼^th^ strength Hoagland solution^36^. Well-established plants were transferred to pots and further analysed for transgene integration, expression and insecticidal efficacy.

### Molecular analyses for T-DNA integration

Total genomic DNA was isolated from tender leaves of transgenic and non transgenic plants^37^. PCR analysis was carried out to amplify gene fragments of transgenes using standardized primers and conditions (Supplementary Fig. S1b). The PCR reaction mixture (25 µl) contained 1 U Taq DNA polymerase (Bangalore Genei, Bangalore), 1 × assay buffer (10 mM pH 9.0 Tris HCl, 50 mM KCl, 1.5 mM MgCl_2_, 0.01% gelatin), 150 µM of each dNTPs, 0.5 µl of each forward and reverse primer at a final concentration of 0.25 µM and 100 ng template DNA. The DNA extracted from non transgenic plant was used as a negative control, while pBinAR vector was used as a positive control and the reaction mix without DNA as water blank. The PCR reaction profile comprised of 30 cycles, with strand separation at 94 °C for 1 min, annealing at 58 °C for 1 min and extension at 72 °C for 1 min. The program was extended at 72 °C for 10 min. The products were electrophoresed on a 0.8% agarose gel, stained with ethidium bromide and visualized under ultraviolet light^38^.

### Southern analysis

For analysis of copy number by genomic Southern analysis, purified genomic DNA (15 µg) from transgenic and non transgenic plants was digested overnight with *Hind*III (NEB high fidelity, NEB), that has a single restriction site in the T-DNA. The digested DNA samples were electrophoresed on a 0.8% agarose gel in TAE buffer and blotted onto a positively charged nylon membrane (Millipore India private Ltd.). A 640 bp PCR product of *cry2Aa* was labeled using DIG PCR labeling kit. Hybridization and washing was carried according to manufacturer’s instructions (Roche Holding AG) and the membrane was incubated for 18 h in dark, wrapped and exposed to an X-ray film for 30 min in dark.

### Analysis of transgenic plants for *cry2Aa* transcript accumulation

#### Sq-PCR

Total RNA^39^ was isolated from the leaves of both transgenic and non transgenic plants and 1 µg was reverse transcribed to single stranded cDNA according to the manufacturer’s instructions (Agilant Accuscript high fidelity kit; www.agilant.com). To evaluate transcript accumulation, 1 µl of the cDNA mix was used as a template for the amplification of 250 bp *cry2Aa* fragment, 416 bp *18srRNA* gene fragment and 200 bp *actin* gene fragment, following initial denaturation at 95 °C for 5 min and 35 cycles of 95 °C for 30 sec, 55 °C for 30 sec, 72 °C for 30 sec and a final extension of 72 °C for 5 min. The products were later visualized on a 0.8% agarose gel.

#### qPCR

Total RNA was isolated from leaves of both transgenic and non transgenic plants (Spectrum^™^, Sigma-Aldrich). Yield and integrity of RNA was assessed using a Nanodrop Micro Photometer (Thermo Scientific) and by agarose gel electrophoresis respectively. cDNA was synthesized from 1 µg total RNA according to manufacturer’s instructions (SuperScript^®^VILO^™^, Invitrogen). Primers to amplify *cry2Aa* gene and *Initiation* *factor* *4α*^40^ (*IF4α*-a house keeping gene to normalize the data) were used to set up qPCR reaction (Supplementary fig. S1b). PCR program consisted of 3 min initial denaturation at 95 °C followed by 40 cycles of 95 °C for 30 sec, 55 °C for 30 sec and 72 °C for 20 sec. Melting curve was analysed at 95 °C for 30 sec followed by 60 °C for 30 sec and 95 °C for 30 sec and used for ascertaining the variations in gene expression among the transgenic events *vis-à-vis* non-transgenic.

### Analysis of Cry2Aa expression in the transgenic events

#### Dot blot analysis

A high throughput protein dot blot analysis was performed to analyze the expression of Cry2Aa protein in the transgenic *vis-à-vis* non transgenic plants. About 100 mg of fresh leaf tissue from the third fully expanded leaf was used for extraction in 50 mM carbonate buffer (pH 9.0). Following quantification using Bradford assay^41^, 10–15 µg total protein (transgenic as well as non transgenic plants) was mixed with 25 µl of 0.4% SDS, volume made up to 200 µl with carbonate buffer and loaded onto an activated nitrocellulose membrane (activated by dipping in carbinol for 30 sec). Dot blot set up was prepared by first placing 4 wet Whatmann paper no. 3 followed by the charged nitrocellulose membrane. Samples were loaded in wells and 20 Kpa suction pressure was applied until protein solution drained out of the wells onto the membrane. The blot was later dried and stored at 4 °C until further development.

#### Western blot analysis

Total protein was isolated from 100 mg of leaf tissue from both transgenic and non transgenic plants using 50 mM phosphate buffer (pH 7.0). Protein was quantified by Bradford’s assay and 20–25 µg was subjected to PAGE and blotted onto nitrocellulose membrane by using transfer buffer (1.5 g Tris, 7.2 g glycine and 100 ml methanol made up to 500 ml with distilled water).

Development of both western and dot blots was initiated by blocking non-specific binding using NAP blocker (Non-animal protein blocker, G Biosciences) followed by initial hybridization with 1:3000 dilution of primary antibody (specific to Cry2Aa; procured from Amar immunodiagnostics, Hyderabad, India) and subsequently with 1:6000 dilution of HRP conjugated secondary antibody. The blots were washed thrice with 1 × PBST after each hybridization followed by addition of TMB (3,3′,5,5′-Tetramethylbenzidine, Promega) substrate for colour development. Dot blots were analyzed and quantified via ImageJ (http://imagej.nih.gov/ij) software following online instructions.

### Bioefficacy of the *cry2Aa* gene in pigeon pea pods and leaves

Efficacy of the transgenic *vis-à-vis* non transgenic plants against *H*. *armigera* was assessed in both leaves and pods. *In vitro* bioassays with the leaves were conducted in all the generations (T_1_ to T_3_) whereas pod bioassay was conducted in T_3_ generation. Fully expanded trifoliate leaves from 45–60 days old plants and 2 week old tender pods were excised and the leaf petioles/the pod stalks were covered with cotton to maintain moisture in the bioassay plates. Over each trifoliate leaf, 10 neonate larvae were released whereas three 2^nd^ instar larvae were released on the pods. Two replicates from each plant were maintained for each treatment. Larval mortality, growth and extent of damage on the plant tissue were recorded at 24, 48, 72, and 96 h after release of larvae.

The experiments were conducted in a completely randomized design (CRD). For statistical analyses, the resultant bioefficacy data was subjected to analysis of variance (ANOVA) followed by mean separation by the Student–Newman–Keuls’ test (P = 0.05). Graphs and regression models were built using R language in R studio version 1.0.136. For regression model, the observations were split into train and test sets with 2/3 ratio. Chi-square (χ^2^) test was performed in the T_3_ generation to assess the observed segregation ratio (3:1) for the transgene^42^ based on the performance in the pod bioassay.

## Electronic supplementary material

Supplementary information
