# Supplementary material for: Expression of Cry2Aa, a Bacillus thuringiensis insecticidal protein in transgenic pigeon pea confers resistance to gram pod borer, Helicoverpa armigera
Source: Sci Rep. 2018 Jun 11;8:8820. doi: 10.1038/s41598-018-26358-9 (PMC5995972; doi:10.1038/s41598-018-26358-9)
Supplement: Supplementary file 1 — Supplementary information [file 41598_2018_26358_MOESM1_ESM.pdf]

Expression of Cry2Aa, a *Bacillus thuringiensis* insecticidal protein in transgenic pigeon pea confers resistance to gram pod borer, *Helicoverpa armigera*

Shweta Singh#, Nikhil Ram Kumar#, R. Maniraj#, R. Lakshmikanth, K. Y. S. Rao, N. Muralimohan, T. Arulprakash, K. Karthik, N. B. Shashi Bhushan[3], T. Vinutha[1], Debasis Pattanayak, Prasanta K Dash, P. Ananda Kumar[2] and Rohini Sreevathsa*

(a)

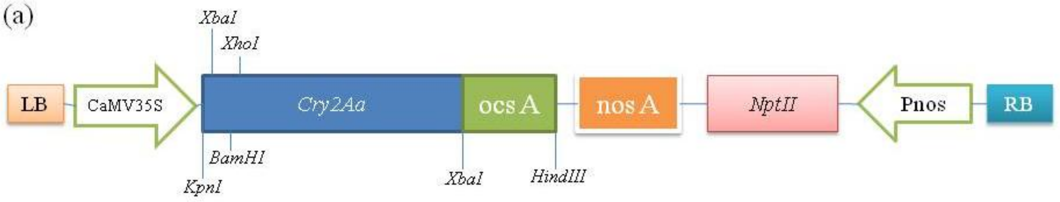

(b)

Primer name	Nucleotide sequence	Fragment length
Cry2Aa FP (PCR)	TCAGGGACGTGATCCTCAACGC	1000 bp
Cry2Aa RP (PCR)	TCGCCCTGGTTGCCGAACTT	
Cry2Aa FP (PCR)	ACACCTACCAGACTGCCTTC	640 bp
Cry2Aa RP (PCR)	AGGTCCTCGTTCCTGATCAC	
NptII FP (PCR)	CCGGAATTCATGATTGAACAA	750 bp
NptII RP (PCR)	CCCAAGCTTCAGAAGAACTC	
Cry2Aa FP (RT)	GCCGTTCCTCTACTCTCTCT	350 bp
Cry2Aa RP (RT)	CGGTGGAGCAGTTGAAGTTA	
18SrRNA FP (RT)	CGCGCAAATTACCCAATCCTGAC	416 bp
18SrRNA RP (RT)	TCCCGAAGGCCAACGTAAATAGGA	
Actin FP (RT)	CCTGAGGTCCTTTTCCAACC	200bp
Actin RP (RT)	GGATTCCGGCAGCTTCCATT	
Cry2Aa FP (qRT)	AGTACCAGTCCCTCATGGT	120 bp
Cry2Aa RP (qRT)	TCACCTGGAAGAGAGAGTAGAG	
IF4 FP (qRT)	GCCGAGATCACACAGTCTCA	95 bp
IF4 RP (qRT)	ACCACGAGCCAAAAGATCAG	

Supplementary figure S1: (a) Vector map of the binary vector pBinAR harboring *cry2Aa* and *nptII* used to generate transformants; (b) List of various primers used in the study

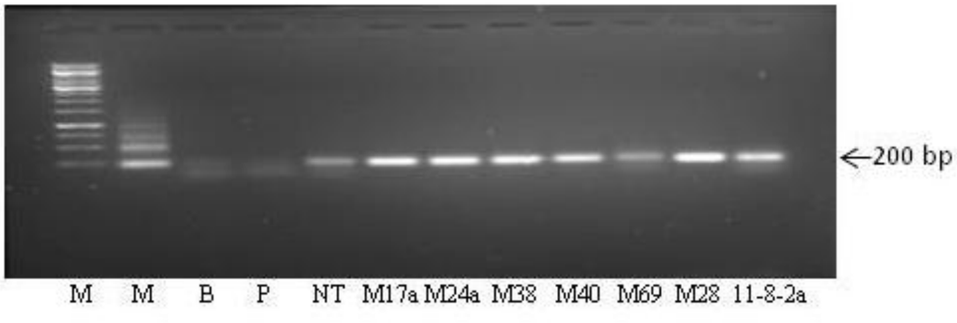

Supplementary figure S2 (original gel picture of Fig. 6c [ii]): Semi quantitative RT-PCR analysis of the selected transgenic plants *vis a vis* non transgenic for the detection of 200 bp actin transcript (M: ladder; B: template blank; NT: non transgenic; P: plasmid);
